# Evaluation of Natural Materials as Exogenous Carbon Sources for Biological Treatment of Low Carbon-to-Nitrogen Wastewater

**DOI:** 10.1155/2015/754785

**Published:** 2015-10-01

**Authors:** Juan Ramírez-Godínez, Icela Beltrán-Hernández, Alejandro Álvarez-Hernández, Claudia Coronel-Olivares, Elizabeth Contreras-López, Maribel Quezada-Cruz, Gabriela Vázquez-Rodríguez

**Affiliations:** ^1^Centro de Investigaciones Químicas, Universidad Autónoma del Estado de Hidalgo, Ciudad Universitaria, Km. 4.5 Carretera Pachuca-Tulancingo, 42067 Mineral de la Reforma, HGO, Mexico; ^2^Universidad Tecnológica de Tecámac, Km. 37.5 Carretera México-Pachuca, Sierra Hermosa, 55740 Tecámac, MEX, Mexico

## Abstract

In the bacterial processes involved in the mitigation of nitrogen pollution, an adequately high carbon-to-nitrogen (C : N) ratio is key to sustain denitrification. We evaluated three natural materials (woodchips, barley grains, and peanut shells) as carbon sources for low C : N wastewater. The amount of organic matter released from these materials to aqueous media was evaluated, as well as their pollution swapping potential by measuring the release of total Kjeldahl nitrogen, N-NH_4_^+^, NO_2_^−^, and NO_3_^−^, and total phosphorous. Barley grains yielded the highest amount of organic matter, which also showed to be the most easily biodegradable. Woodchips and peanut shells released carbon rather steadily and so they would not require frequent replenishment from biological reactors. These materials produced eluates with lower concentrations of nutrients than the leachates from barley grains. However, as woodchips yielded lower amounts of suspended solids, they constitute an adequate exogenous source for the biological treatment of carbon-deficient effluents.

## 1. Introduction

Anthropogenic eutrophication is a major water pollution problem worldwide. Overenrichment of nutrients (nitrogen and phosphorus) increases the production of biomass in aquatic systems, thereby impairing the water quality and threatening the natural balance of these ecosystems. Agricultural runoff, livestock operations, aquaculture, industry (e.g., food processing facilities and pulp and paper mills), sewage treatment plants, and fossil fuel combustion are the major sources of nutrient pollution [1].

The mitigation of nitrogenous pollution mostly relies on biological treatments based on the well-known route ammonification-autotrophic nitrification-heterotrophic denitrification. In these processes, the carbon-to-nitrogen (C : N) ratio is a key design parameter. Although an approximate 10 : 1 ratio (measured as COD/TKN) is frequently recommended, some authors suggest values as high as 20 : 1 or 30 : 1 [2]. However, at excessively high C : N ratios heterotrophic bacteria can outcompete nitrifying microorganisms [3], whereas low C : N ratios limit denitrification and can cause the accumulation of NO_2_^−^ in total nitrogen removal processes [4].

Many of the aforementioned pollution sources generate effluents with disproportionately high contents of nitrogen. For instance, wastewater from the optoelectronics industry is rich in organic nitrogen because ethanolamine and tetramethyl ammonium hydroxide are used in the manufacturing process [5]. Intensive aquaculture systems tend to generate effluents enriched in NH_4_^+^ [6, 7], as well as petrochemical, pharmaceutical, fertilizer, and food industries [8]. Stainless steel manufacturing processes generate wastewater with nitrate concentrations ranging from 500 to 1000 mg N-NO_3_^−^/L [9], while in agriculturally impacted groundwater a range of 1-2 mg N-NO_3_^−^/L is expected [10]. In the two last cases, hardly any organic matter is found.

Any unbalanced C : N wastewater requires the addition of exogenous carbon sources. A wide variety of compounds has been employed for this purpose, such as sugars, organic acids, alcohols, and oils [11], though methanol appears to be the most common [9]. Recently, solid materials have received more attention, and consequently cornstalks [11], wood by-products (e.g., sawdust and woodchips) [12], wheat straw [13], compost [14], and starch-based biodegradable polymers [15], among other substrates, have also been used as external carbon donors.

When selecting a carbon source, several aspects must be considered, such as its cost, denitrification rate, handling safety, and potential release of toxic compounds. In fact, the costs of the carbon donor and the sludge management are key, as they account for more than 50% of the overall wastewater treatment cost [9]. The performance of the treatment is often hindered by the export of excessive amounts of dissolved organic carbon from the source [16]; consequently, in order to guarantee a proper dosage of the material, the amount of organic matter leached must be assessed. Another important aspect of the carbon source to take into account is its pollution swapping potential, which is the increase in one pollutant concentration as a result of an action implemented to remove another pollutant [17]. Nevertheless, even if some natural materials have been extensively used as carbon donors, their potential of pollution swapping is rarely quantified.

We evaluated three economical, natural materials (woodchips, grains of feed barley, and peanut shells) as potential carbon sources for low carbon-to-nitrogen wastewater. In leaching tests, we measured the amount of organic matter released from these materials in aqueous media, as well as their cross pollution potential in terms of release of nitrogenous compounds (total Kjeldahl nitrogen, N-NH_4_^+^, NO_2_^−^, and NO_3_^−^) and total phosphorous (TP). In addition, the chemical characteristics of the raw materials and the leached organic matter were studied by elemental analysis and infrared spectroscopy.

## 2. Materials and Methods

### 2.1. Natural Materials

Woodchips, grains of feed barley (*Hordeum vulgare* L.), and peanut (*Arachis hypogaea*) shells were studied as potential carbon sources. Woodchips were obtained from untreated pine (*Pinus sylvestris*). According to screen analysis, 85.5% of the woodchips were retained over a 2 mm mesh sieve, while 10.4% passed through a 2 mm mesh sieve but were retained over a 1 mm mesh sieve. The remaining fraction (4.1%) passed through a 1 mm mesh sieve but was retained over a 0.6 mm mesh sieve. Barley grains (0.6 ± 0.05 cm) and peanut shells (3.2 ± 0.47 cm) were obtained, respectively, from Apan and Temascalapa, both in the State of Hidalgo, Mexico. All foreign matter (such as stones, dust, or stalks) and damaged kernels were removed from barley by hand.

Elemental composition and Fourier Transform Infrared (FT-IR) analyses were made on 1.5–3.0 mg samples of the natural materials previously ground to a homogeneous fine powder and dried at 105°C for 24 h using a 2400 Series II CHNS Elemental Analyzer and a Spectrum GX FT-IR spectrometer (both from Perkin-Elmer, Waltham, MA, USA), respectively. The IR spectra were obtained from KBr pellets (1 : 100 weight ratio of sample/KBr). The spectrometer was set to scan from 4000 to 400 cm^−1^.

### 2.2. Batch Leaching Tests

Leaching tests were performed in batch mode at a solid-to-liquid ratio of 50 g/L. The materials were washed with distilled water and air-dried. Samples of the natural materials were added separately to 1 L of distilled water in glass flasks. The flasks were purged for 10 min with N_2_, sealed, and then placed under agitation on an orbital shaker (Polyscience, USA) at 120 rpm and at room temperature for 50 days. Periodically, 100 mL samples of the supernatants were taken from each flask and maintained at 4°C until analysis.

After 50 days, the supernatants were completely withdrawn from the flasks, filtered, and separated in about 1 mL portions to be lyophilized by continuous freeze drying (Freeze Dry System, Freezone 4.5, Labconco, Kansas City, MO, USA) at –50°C and 133 × 10^−3^ mBar. Lyophilized leachates were analyzed for elemental composition and FT-IR spectroscopy as for raw natural materials (Section 2.1).

### 2.3. Chemical Analyses of Aqueous Leachates

Except where indicated otherwise, the analyses were made according to the* Standard Methods for the Examination of Water and Wastewater* [18]. Chemical oxygen demand (COD) was analyzed spectrophotometrically at 600 nm after digestion of the samples with K_2_Cr_2_O_7_/H_2_SO_4_ (method 5520). Ultraviolet absorbance at 245 nm (UV_254_) was measured in a 1 cm quartz cell (method 5910). Dissolved organic carbon (DOC) was measured with an infrared analyzer (Shimadzu, Japan). BOD_5_ was determined in the OxiTop measurement system (WTW, Germany). For measuring TKN, the samples were digested at 400°C and distilled in a Gerhardt Vapodest 20 (Germany) unit to transform organic nitrogen into ammonium ions, which were further assessed by titration with 0.01 N HCl. N-NH_4_^+^ was assessed by the phenate method (4500-NH_3_). The 4500-NO_2_^−^ and 4500-P F methods were used for measuring N-NO_2_^−^ and TP, respectively. Finally, the content of N-NO_3_^−^ was determined by the phenoldisulfonic acid method [19].

## 3. Results and Discussion

### 3.1. Chemical Composition of the Raw Natural Materials

The elemental analysis of the raw materials showed that carbon and hydrogen contents were 50.56% and 6.36% for woodchips, 48.01% and 5.31% for barley grains, and 44.49% and 6.47% for peanut shells, respectively. Nitrogen was only detected in barley samples, where it accounted for 0.97%. This value is lower than those usually reported in the literature (e.g., 1.27–2.01%) [20]. In cereals, the N content is directly associated with the total protein content, which is about 8–13% in feed barley [21]. Although an N content of about 2.3% has been found in peanut shells [22], also mostly related to proteins, this element could not be detected in our samples.

The FT-IR spectra of the three raw materials showed almost identical main signals. A broad band centered at 3400 cm^−1^ was detected, which is characteristic of the O–H stretching. A signal observed at 2920 cm^−1^ was attributed to C–H stretching, while the signal found at 1650–1640 cm^−1^ is characteristic of bending motions of absorbed water. A complex signal detected between 1470 and 1350 cm^−1^ was assigned to C–H bending and scissoring motions, and finally the strong signal at 1030–1020 cm^−1^ comes from C–O stretching. These signals are very well in agreement with the cellulosic nature of these natural materials. Indeed, cellulose accounts for about 34–48% of the mass of pinewood [23], 3.1–4.4% of barley grains [24], and about 35.2% of peanut shells [25].

### 3.2. Chemical Composition of the Lyophilized Leachates

During leaching tests, submerged plant material releases soluble compounds due to the breakdown of the vacuoles of plant cells by the physical action of water [26]. The analysis of both the elemental composition and the FT-IR spectra of the lyophilized 50-day leachates provided information about the compounds solubilized preferentially from the solid matrix.

Woodchips yielded the leachate with the highest carbon percentage (50.4%), followed by barley grains (26.7%) and peanut shells (21.7%). Concerning the nitrogen content, barley leachate presented the highest percentage (1.9%), which is consistent with the results of the elemental analysis carried out on the raw material. Peanut shells released an eluate with 1.5% of nitrogen, even though this element was not detected in the solid material by elemental analysis. In the leachate from woodchips, this nutrient was not found.

The FT-IR spectra of the woodchip and barley leachates showed no substantial change from their corresponding raw material. However, the FT-IR spectrum of the leachate of peanut shells showed a strong band at 1400 cm^−1^ that, along with a weak sharp band at 835 cm^−1^, indicated the presence of nitrate. As this band was not detected in the raw material, it was hypothesized that the proteins of peanut shells were hydrolyzed and further ammonified to yield ammonium, which could be finally oxidized to nitrates by nitrifying bacteria. The leaching tests were conducted by preventing the entrance of oxygen into the flasks; yet, aerobic or microaerobic conditions could have been established, leading to nitrification. Although barley grains have higher protein content than peanut shells and the aforementioned bacterial processes could have occurred in barley leachate too, the FT-IR spectrum did not show the presence of nitrates. The results of both analyses suggest that barley grains and peanut shells could be inadequate carbon sources, because they might lead to a significant cross pollution due to the leaching of nitrogen compounds.

### 3.3. Leaching Kinetics of Organic Matter

**Figure 1:**
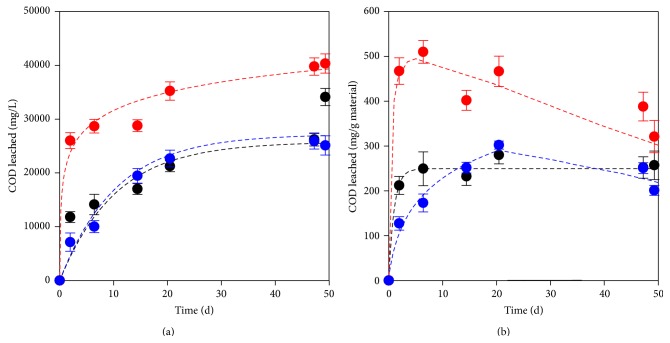
Leaching of organic matter measured as (a) concentration of COD and (b) mass of COD released per gram of natural material. Red bullet: barley grains, blue bullet: peanut shells, and black bullet: woodchips.

Figure 1(a) shows the course of the concentration of organic compounds measured as COD in the eluates. In this figure, the typical biphasic curves displayed by natural materials in aqueous leaching tests are worth noting. In our assays, an appreciable concentration of organic matter was released from the three materials in the first 20 days; it accounted for 54, 89, and 86% of the COD content measured at the end of the leaching tests of woodchips, peanut shells, and barley grains, respectively. After 20 days, additional COD was still released but at a slower pace. This biphasic behavior has been also reported for other natural materials, such as wild sugar cane (*Saccharum spontaneous*) [26] and pine sawdust [27]. Accordingly, the degradation of submerged organic materials seems to start with a leaching phase, which is followed by a hydrolysis phase characterized by the breakdown of the released macromolecules into simpler compounds.

Figure 1(b) shows the COD released per gram of each natural material. These data were obtained from mass balances taking into account the volume of medium extracted during samplings. For the leaching of COD from woodchips, the biphasic behavior was still observed, but the slow phase started earlier (after only two days). This is consistent with the results of Svensson et al. [27], who reported reaching an equilibrium in the leaching of organics from pine sawdust after 50 hours. The amount of COD released per gram of woodchips in the first two days of testing represented about 94% of the total COD released after 50 days. In the eluates of peanut shells and barley grains, after reaching a maximum release, a further consumption of COD was noticed. The depletion of the organic matter leached from barley started after the 6th day of testing; for the eluate of peanut shells, it started after 20 days. This implies that the leachate of barley grains is more biodegradable than that of peanut shells. Barley grains are mostly constituted by easily biodegradable macromolecules (carbohydrates: 60–80%; proteins: 13–16%; lipids: 1-2%) [28] and they have a low content of cellulose (a slowly biodegradable polymer); consequently, the aqueous leachate obtained from this material is expected to be also easily assimilable.

**Figure 2:**
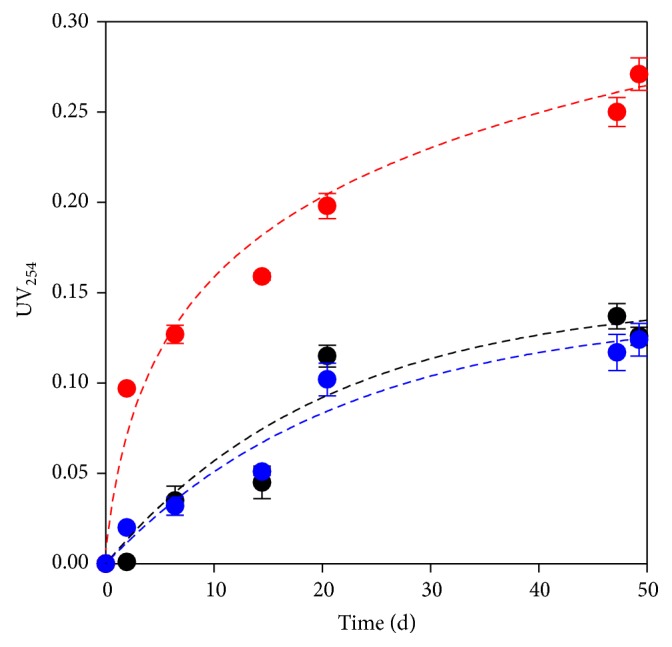
Organic matter (measured as UV_254_) released from natural materials. Red bullet: barley grains, blue bullet: peanut shells, and black bullet: woodchips.

The release of organic matter was also assessed in terms of UV_254_ (Figure 2), because some organic compounds abundantly found in plants, such as lignin, tannins, and other phenolics, strongly absorb UV radiation [29]. Thus, UV_254_ was considered a surrogate for the organic matter released by the plant materials, which was supported by the strong correlation (*r* ^2^ > 0.84) found between our measurements of COD and UV_254_. Other studies have also reported this correlation in wetland effluents [30]. However, as UV_254_ is related to aromatics, it is more closely associated with persistent organic matter (e.g., the humic fraction of natural organic matter) rather than total organic content.

As for COD, barley was the material that released the highest amount of aromatic organic matter (Figure 2). In fact, barley grains are good sources of phenolic compounds (between 450 and 1346 mg/g) [31] such as benzoic and cinnamic acids, flavonoids, tannins, coumarins, and resorcinols, which all can contribute to the UV_254_ value. In woodchips and peanut shells, the major source of phenolics is likely to be lignin, which represents 44.9% [32] and 36.5% [33] of these materials, respectively. In barley grains, lignin only constitutes 2.9% [34].

**Figure 3:**
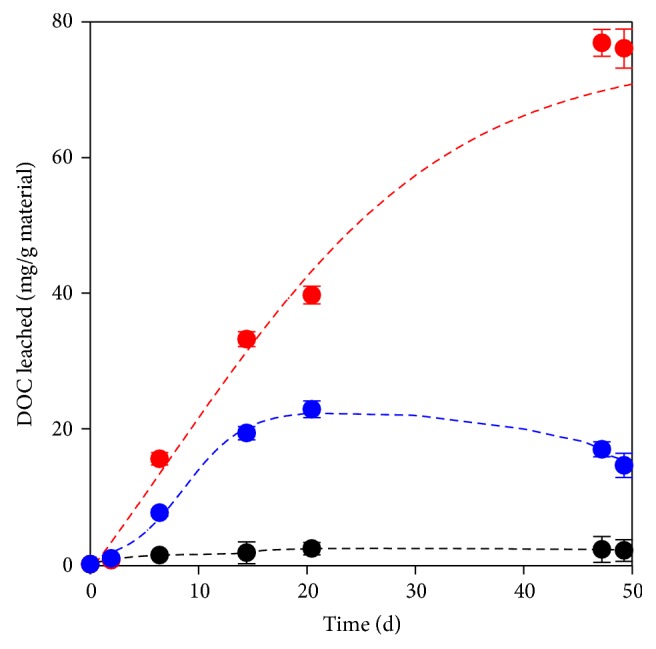
Organic matter (measured as DOC) released per gram of natural material. Red bullet: barley grains, blue bullet: peanut shells, and black bullet: woodchips.

If the DOC is considered, barley was also the material that released the highest amount (Figure 3). Nonetheless, for this material, a linear DOC leaching was observed, rather than the aforementioned biphasic behavior. After 50 days of testing, the DOC accumulated was 2.0, 14.6, and 76.1 mg/g of woodchips, peanut shells, and barley grains, respectively. The accumulation of DOC released from woodchips was considerably smaller than the value (i.e., 45 mg/g) previously reported for pine wood chips after only two days of leaching [27], probably due to variations in the mean size of chips or in the solid/liquid ratios used in the leaching tests. The DOC released in our assays accounted for only 0.4, 0.8, and 17.1% of the initial carbon content measured by the elemental composition analysis of woodchips, peanut shells, and barley, respectively. For woodchips with several particle sizes (0.60, 1.18, and 4.75 mm), a prior study [35] had reported an organic carbon release of 1.1, 0.80, and 0.60% after 7 days, respectively. Even though the leaching periods are different, this last value is consistent with our results, corresponding to woodchips with a mean size higher than 2.0 mm (85.5%). It has been advocated that woody materials are used more steadily than other natural sources because their carbon is not rapidly depleted [17]. In this way, they do not require frequent replenishment from biological reactors.

BOD_5_ was measured only in the samples taken after 30 days of testing (data not shown). For these samples, the BOD_5_/COD ratios of the leachates of woodchips, peanut shells, and barley grains were 0.15, 0.49, and 0.42, respectively. A BOD_5_/COD ratio very similar (0.14) to the first value was reported for an old wood waste leachate [36]. According to these ratios, the organic matter leached from woodchips is less easily biodegradable than the organics leached from both peanut shells and barley. This is in agreement with the consumption of the COD signaled before for the eluates from peanut shells and barley grains (Figure 1(b)). Wood leachates contain a mixture of hemicellulose, lignin, tannins, and fatty acids, among other compounds [36]. The high molecular weight of some of these compounds could explain the low biodegradability of the leachates. In addition, it has been reported that wood leachates are toxic due to the presence of tannins, lignin, tropolones, terpenes, and lignans [36]; however, the performance of wood-like materials as carbon donors in biological treatments has been widely experienced [4, 11, 12].

### 3.4. Leaching Kinetics of Nutrients

**Figure 4:**
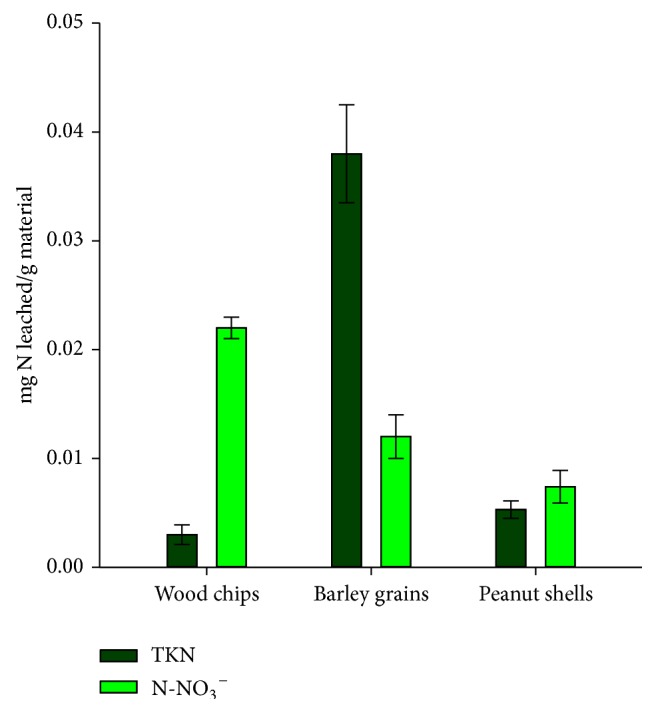
Nitrogenous species leached per gram of natural material after 50 days of testing.

Besides organic carbon, submerged plant materials are likely to release inorganic compounds.  Figure 4 shows the amount of nitrogenous species released at the end of the tests (any concentration of nitrites was detected in the eluates).

After 50 days, barley grains yielded the highest amount of TKN among the materials tested, about 0.038 mg N/g. This can be attributed to the high protein content of this cereal, which is thereafter released into water. The concentrations of TKN released by woodchips (0.003 mg N/g) and peanut shells (0.005 mg N/g) are considerably lower than that produced by barley grains, because wood is composed mainly of organic molecules lacking nitrogen, such as lignin and polysaccharides. That is why nitrogen could be detected only in barley samples by the elemental analysis of the materials (Section 3.1). In fact, the poor N content of wood is one of the main causes of its environmental persistence, as nitrogen is the limiting growth factor for fungi required for exoenzyme production [37].

Even though the FT-IR analysis only evidenced nitrate in the leachate of peanut shells, we detected this ion in all the three eluates. As just stated, wood has a low content of N, and so the nitrates found in the leachate might come from some kind of foreign material. In the eluates of barley grains and peanut shells, nitrates were more likely to arise from the microbial oxidation of the organic nitrogen of these materials.

Peanut shells produced the least concentrations of total nitrogenous species, due to the low content of protein of this material (6-7%) [38]. Peanut shells are composed mainly of fiber (60–67%) [38] and their content of lignin (36.5%) [33] is even higher than that of most hardwoods and softwoods [39]. However, peanut shells disintegrate easily in aqueous media and produce high levels of suspended solids and turbidity (data not shown). Therefore, their use in biological reactors would require continuous replacement from the treatment system and would lead to a considerable pollution swapping.

**Figure 5:**
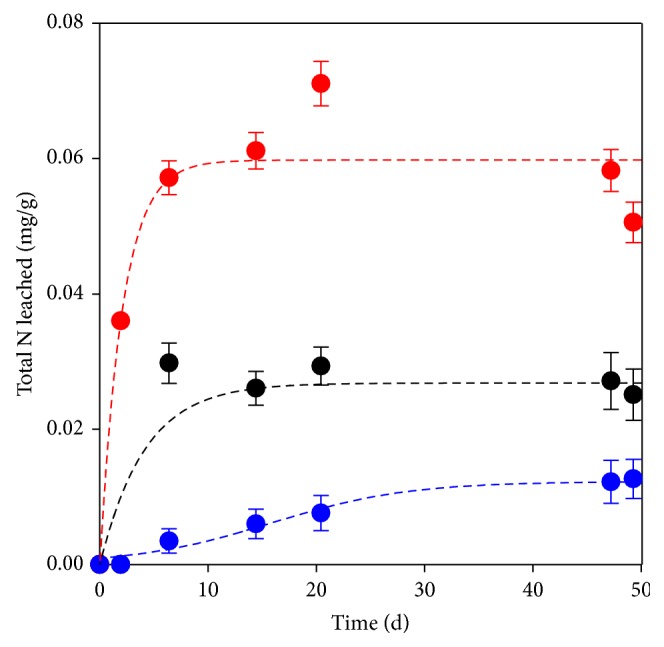
Total nitrogen released per gram of natural material. Red bullet: barley grains, blue bullet: peanut shells, and black bullet: woodchips.

When all the nitrogenous species (i.e., TKN plus nitrates) are considered, barley is the material that releases the highest amount of this nutrient to the liquid media (Figure 5). At the end of the leaching tests, the materials had released 0.05, 0.025, and 0.013 mg N per gram of barley grains, woodchips, and peanut shells, respectively. In the case of barley grains, for which an initial N content of 0.97% had been determined by elemental composition analysis, the release of N to the liquid media only corresponded to 0.7% of the initial input of this element.

**Figure 6:**
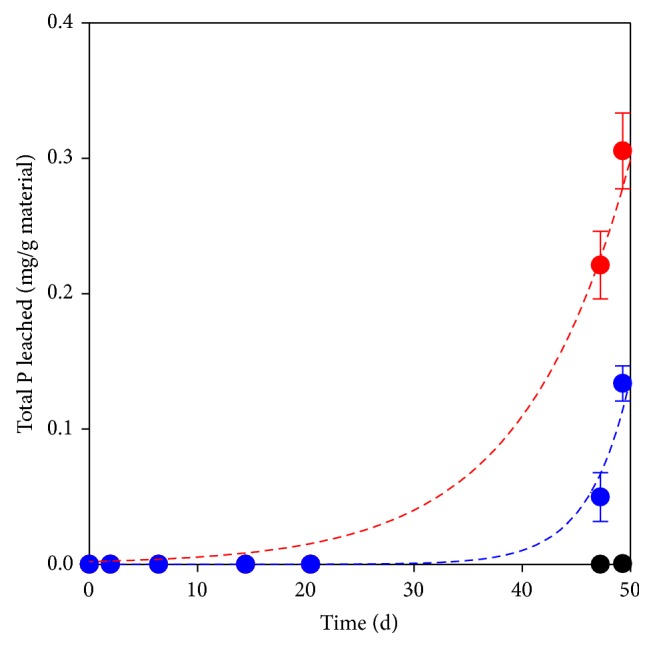
Total phosphorus (TP) released per gram of natural materials. Red bullet: barley grains, blue bullet: peanut shells, and black bullet: woodchips.

The release of total phosphorus from the three materials is shown in Figure 6. Woodchips released barely detectable quantities of phosphorus throughout the experiment, whilst barley grains and peanut shells yielded about 0.30 and 0.13 mg TP per gram of material, respectively, at the end of the leaching tests. If the P contents of barley grains and peanut shells reported in the literature (0.37% [24] and 0.025% [22], resp.) are considered, it can be estimated that only 2.2% and 16%, respectively, of the initial input of this element were solubilized from the solid matrix after 50 days.

In this study, the release of organic matter and nutrients from submerged natural materials was studied quantitatively. Its main practical implication is the design of biological processes based on the use of these materials for treating low C : N wastewater with a stoichiometric approach. In this way, the release of carbon could be controlled to sustain the activity of all the bacterial groups involved in total nitrogen removal. For instance, the control of both the C : N ratio and the nitrate recycling ratio at optimal levels augmented the total nitrogen removal in biological aerated filters by enhancing the denitrifying activity [40]. In another study [41], the C : N ratio was controlled by a step-feeding strategy, which resulted in the increase of the total nitrogen removal in constructed wetlands. Similar results might be obtained in processes involving the natural materials studied here as external carbon sources for low C : N wastewater.

## 4. Conclusions

We evaluated three solid natural materials, woodchips, peanut shells, and barley grains, as potential carbon donors for the biological treatment of wastewater with an unfavorably low carbon-to-nitrogen ratio. On one hand, the analyses of the leachates indicated that woodchips and peanut shells are suitable carbon sources, as they release organic matter but lesser amounts of nutrients than barley grains. On the other hand, the organic matter released from woodchips is less easily biodegradable than that released by peanut shells and barley grains. The main drawback of peanut shells is that they disintegrate easily, thereby increasing the turbidity and the content of suspended solids of leachates. In conclusion, woody materials may be considered as adequate and economical carbon donors that minimize cross pollution in wastewater treatment.
